# Na/K-ATPase signaling mediates miR-29b-3p regulation and cardiac fibrosis formation in mice with chronic kidney disease

**DOI:** 10.1371/journal.pone.0197688

**Published:** 2018-05-18

**Authors:** Christopher A. Drummond, Xiaoming Fan, Steven T. Haller, David J. Kennedy, Jiang Liu, Jiang Tian

**Affiliations:** 1 Department of Medicine at the University of Toledo, Toledo, OH, United States of America; 2 Joan C. Edwards School of Medicine, Marshall University, Huntington, WV, United States of America; Universidade Federal do Rio de Janeiro, BRAZIL

## Abstract

The Na/K-ATPase is an important membrane ion transporter and a signaling receptor that is essential for maintaining normal cell function. The current study examined the role of Na/K-ATPase signaling in regulating miR-29b-3p, an anti-fibrotic microRNA, in a mouse chronic kidney disease (CKD) model (5/6^th^ partial nephrectomy or PNx). The results showed that CKD induced significant reduction of miR-29b-3p expression in the heart tissue by activation of Src and NFκB signaling in these animals. To demonstrate the role of Na/K-ATPase signaling, we also performed the PNx surgery on Na/K-ATPase α1 heterozygous (α1+/-) mice, which expresses ~40% less Na/K-ATPase α1 compared to their wild type littermates (WT) and exhibits deficiency in Na/K-ATPase signaling. We found that CKD did not significantly change the miR-29b-3p expression in heart tissue from the α1+/- animals. We also found that CKD failed to activate Src and NFκB signaling in these animals. Using isolated cardiac fibroblasts from α1+/- mice and their WT littermates, we showed that ouabain, a specific Na/K-ATPase ligand, induces decreased miR-29b-3p expression in fibroblasts isolated from WT mice, but had no effect in cells from α1+/- mice. Inhibition of NFκB by Bay11-7082 prevented ouabain-induced miR-29b-3p reduction in WT fibroblasts. To further confirm the *in vivo* effect of Na/K-ATPase signaling in regulation of miR-29b-3p and cardiac fibrosis in CKD animals, we used pNaKtide, a Src inhibiting peptide derived from the sequence of Na/K-ATPase, to block the activation of Na/K-ATPase signaling. The result showed that pNaKtide injection significantly increased miR-29b-3p expression and mitigated the CKD-induced cardiac fibrosis in these animals. These results clearly demonstrated that Na/K-ATPase signaling is an important mediator in CKD that regulates miR-29b-3p expression and cardiac fibrosis, which provides a novel target for regulation of miR-29b-3p in CKD. We also demonstrate that antagonizing Na/K-ATPase signaling by pNaKtide can reduce organ fibrosis through the stimulation of tissue miR-29b-3p expression.

## Introduction

MicroRNAs (miRNAs) are a group of non-coding RNAs that are 18–25 bp in length. These small RNAs can bind to the targeted mRNAs and inhibit their translation into functional proteins [1–3]. Studies have found a series of miRNAs that specifically target the mRNAs of extracellular matrix proteins and are associated with tissue fibrosis [3–9]. Regulation of miRNAs is important in therapeutic interventions, and strategies have been developed and tested in several disease settings including cardiovascular disease [10–15]. Studies have shown that reduction of miR-29b-3p can cause fibrosis in heart, lung, liver, skin and kidney, while increase of miR-29b-3p prevents tissue fibrosis [7, 8, 16–19]. Cardiac fibrosis is common in cardiac diseases, and accumulation of collagen and other matrix proteins in the extracellular space forms interstitial fibrosis [20–23]. Experimental and clinical data showed that fibrosis increases cardiac stiffness, but reduction of fibrosis may improve cardiac function [24, 25]. Fibrosis could also lead to sudden cardiac death even in conditions that has no cardiac symptoms [26].

Na/K-ATPase is an important cell membrane protein enriched in heart and kidney tissues. The Na/K-ATPase signaling involves Src, Akt, PKC, and other downstream signaling proteins [27]. Our laboratory has previously shown that formation of cardiac fibrosis involves increases in endogenous cardiotonic steroids (CTS) and activation of Na/K-ATPase signaling in the 5/6^th^ partial nephrectomy (PNx) model of chronic kidney disease (CKD) [28–30]. We also found that in isolated cardiac fibroblasts, treatment with ouabain, a Na/K-ATPase ligand, induced decreases in miR-29b-3p and increased collagen expression, whereas blocking the Na/K-ATPase related signaling pathway restores miR-29b-3p levels and mitigated collagen expression in these cells [31]. The current study was designed to test the *in vivo* effect of Na/K-ATPase signaling in regulating the expression of miR-29b-3p and tissue fibrosis in a mouse model of CKD.

## Materials and methods

### Animals

Animal experiments were conducted in accordance with the National Institutes of Health, Guide for the Care and Use of Laboratory Animals under the protocol (IACUC# 106846) approved by the Institutional Animal Care and Use Committee at the University of Toledo. Na/K-ATPase α1 subunit heterozygous (α1+/-) and wild type (WT) mice were generated from C57/Black Swiss mice as previously described [32]. These mice were obtained from Dr. Jerry Lingrel at the University of Cincinnati and maintained in our animal facility. The heterozygous and WT mice were crossed to generate the inbred WT and heterozygous offspring that were used for these experiments. The heart tissue of α1+/- mice contains ~40% less Na/K-ATPase α1 compared to their WT littermates [33, 34]. Adult male mice at 2–3 months of age and weighing between 25-27g were used for this study. All mice were reared under a 12h dark/light cycle, fed standard chow and were provided water *ad libitum*. These conditions were utilized for the entire duration of the experiment.

Male α1+/- mice and their WT littermates were each divided into two groups based on surgical intervention: the first group consisted of sham-operated animals as controls; the second group of animals were subjected to 5/6^th^ partial nephrectomy (PNx) surgery. Following the surgery, each group was further divided into two subgroups: one group received intraperitoneal injection of pNaKtide at the 12^th^ week post-surgery at a dose of 25mg/kg bodyweight, and another group received the same volume of saline as control. PNx surgery was performed as previously described [35]. Briefly, mice were anesthetized with a mixture of 100% oxygen and 2% isoflurane. An incision was made in the left flank on the back, the left kidney was exposed, and the artery supplying the upper pole of the kidney was ligated with a 6–0 silk suture (Coviden, Mansfield, Ma, Cat No: S-1750K) under a high power dissecting microscope. The kidney was then reinserted to the body cavity and the incision was closed. One week later, the right kidney was surgically removed but the renal capsule with the adrenal gland were kept. To alleviate the animals from possible pain due to the surgery, we subcutaneously injected a long-lasting buprenorphine (0.05mg/kg) at 30 min before the surgery.

### Echocardiographic imaging, blood pressure measurement, and organ collection

Echocardiography was performed before surgery as baseline and at the end of the 16^th^ week prior to organ collection using an Acuson Sequoia C512 machine (Siemens) as previously described [35]. Briefly, animals were anesthetized with a mixture of 2% isoflurane and 100% oxygen and were secured to a heated metal platform in a supine position with medical tape on all four extremities. A 15 mHz linear transducer 15L8 (Siemens) was used to acquire images in a shallow left-side position.

Organ collection was done at the end of the 16^th^ week following PNx surgery. Mice were euthanized by anesthesia with a mixture of ketamine and xylazine (100mg/kg and 10mg/kg, respectively) followed by exsanguination. One half of the heart left ventricle or the kidney was immediately placed in a 4% formaldehyde solution for fixation, while the other half was flash frozen in liquid nitrogen and stored at -80°C for later use for biochemical analysis.

### Western blot analysis

Tissue homogenates were prepared by placing left ventricle tissue in ice-cold RIPA lysis buffer (pH 7.0) from Santa Cruz Biotechnology (Cat. No.: SC-24948), followed by homogenization. Aliquots were then made for Western blot analysis or stored frozen at -80°C. Proteins from homogenates were separated by SDS-polyacrylamide gel electrophoresis. The resolved proteins were then electro-transferred to a Nitrocellulose membrane (Fisher Scientific, Hanover Park, IL; Cat. No. 45-004-007) for immunoblotting. Src kinase activation was determined using a primary antibody against phospho-Src at Tyr418 (Fisher Scientific, Cat. No.: 44660G; pSrc). Total Src was probed with a mouse anti-cSrc antibody from Santa Cruz Biotechnology (Cat. No.: SC-8056). Expression of Na/K-ATPase α1 subunit was measured using an anti-Na/K-ATPase α1 antibody from the Developmental Studies Hybridoma Bank at the University of Iowa (Cat. No.: α6F). GAPDH (Santa Cruz; Cat. No. SC-25778) was used as a loading control.

### Histology

Left ventricle sections fixed in 4% formaldehyde solution (pH 7.2) were paraffin embedded and cut into a thickness of 4 μm onto microscopy slides. The tissue sections were deparaffinized with xylene and rehydrated by sequential incubations in ethanol and water. Masson’s Trichrome staining for cardiac fibrosis was conducted on the 4 μm heart tissue sections. Computer aided morphometry was used to quantify the percent area of fibrosis as previously described [28, 35]. For each section, 10 images were randomly taken with a bright-field microscope with a 20X lens. The percentage of blue-color area was measured using ImageJ software. The average of the 10 images from each section was counted as one measurement for statistical analysis.

### Wheat Germ Agglutinin (WGA) staining

Paraffin-embedded left ventricle tissue sections (4 μm in thickness) were deparaffinized and rehydrated in four changes of Xylene, 2 changes of 100% ethanol, 2 changes of 95% ethanol, and 2 changes of 70% ethanol. The tissue sections were then rinsed with tap water and incubated with Oregon Green 488 WGA solution (5 μg/ml) from Life Technologies (Cat. No. W6748) at 4°C overnight. Afterwards, slides were washed three times with PBS. The slides were allowed to air dry, and then mounted with Prolong Gold Antifade Reagent from Life Technologies (Cat. No.: P36930). Eight fluorescent images were randomly taken from each tissue section using an Olympus fluorescent microscope with a 20x lens. From each image, the cross sectional area of 20 cells was measured using ImageJ as previously described [35].

### Isolation, culture and treatment of cardiac fibroblasts

Isolation of cardiac fibroblasts was carried out as previously described [31, 36]. Briefly, hearts of adult male α1 +/- mice or their WT littermates were anesthetized with Ketamine/Xylazine. The heart was cut off from the upper end of the ascending aorta and perfused under sterile conditions with Joklik’s medium from Sigma (Cat. No.: M0518) on a modified Langendorff Apparatus for 5 min. Perfusate was switched to the Joklik’s medium containing 0.1% Collagenase (Sigma; Cat. No.: C0130-1G) and 0.1% bovine serum albumin (BSA) for 25min. After perfusion the heart was cut into small pieces and shaken in the same medium for 30min at 37°C with constant agitation. The resulting cell suspension was centrifuged at 600g for 10min, and the supernatant was centrifuged at 1500g for 15min. The resulting fibroblasts were cultured in Dulbecco’s Modified Eagle Medium from Sigma (Cat. No.: D1152) supplemented with antibiotics and 15% fetal bovine serum (Life Technologies Inc., Cat. No.: 10437–028). The second passage of cells was used for experiments.

### Measurement of NFκB expression and activation

For total expression of NFκB measurement, left ventricle tissue homogenate was analyzed by Western blot using anti-NFκB p65 antibody from Abcam (Cat. No. AB32536). To detect the nuclear fraction of NFκB p65, left ventricle tissue was homogenized and the nuclear and cytoplasmic isolation was performed using a commercial extraction kit (Fisher Thermo, Cat. No. 78835). Western blot was then used to analyze the levels of NFκB p65 in nuclear and cytosol fractions. Lamin B1 was used as a marker and loading control for the nuclear fraction. The Lamin B1 antibody was purchased from Abcam (Cat No: AB16048). The nuclear/cytosol ratio of NFκB was calculated after correction by Lamin B1 and GAPDH, respectively.

### Immunostaining for NFκB P65 in heart tissue

Paraffin-embedded left ventricle tissue sections (4 μm in thickness) were deparaffinized as described above for WGA staining. After blocking with 1% BSA for 1h at room temperature, slides were incubated with anti-NFκB P65 antibody (Abcam, Cat. No: ab16502) at 4°C overnight. The primary antibody was then washed with TBS-T solution for 3 times, followed by incubating with a secondary anti-rabbit antibody conjugated with Alexa 488 for 2h at room temperature. The slides were then incubated with mounting medium containing DAPI for nuclear staining and mounted with coverslip. Fluorescent signals were visualized using a Leica confocal microscope with a 63x oil lens. Five images were taken from each slide. Data from 4–5 animals in each group were analyzed by Two-Way ANOVA with GraphPad software version 7.0.

### RNA Isolation and reverse transcription-quantitative polymerase chain reaction (RT-qPCR)

The left ventricular tissue was homogenized in Qiazol from Qiagen and total RNA was isolated using the miRNeasy mini kit following the protocol provided by the manufacturer. Approximately 250 ng of extracted total RNA was used to synthesize cDNA in the miScript II RT kit (Qiagen, Inc.; Cat. No.: 218160). Quantification of miR-29b-3p expression was performed as previously described [31, 37]. Briefly, Qiagen miScript primer for miR-29b-3p (Cat. No.: MS00005936) was mixed with the cDNA and miScript SYBR Green solution provided in a commercial PCR kit (Cat. No.: 218161, Qiagen, Inc.). RT-PCR was performed on a Qiagen Rotor-Gene Q PCR machine. Calculation of miRNA expression was conducted by comparing the relative change in cycle threshold value (ΔCt) between miR-29-3p and the internal control, RNU6 (Cat No.: MS00033740 from Qiagen). Fold change in expression was calculated for each miRNA using the equation of 2^-ΔΔCt^.

### Statistical analyses

Data are presented as Mean ±Standard Error of the Mean (SEM) and analyzed using One-way or Two-way ANOVA where they are appropriate.

## Results

### Na/K-ATPase is involved in the regulation of miR-29b-3p and cardiac fibrosis in CKD animals

**Fig 1:**
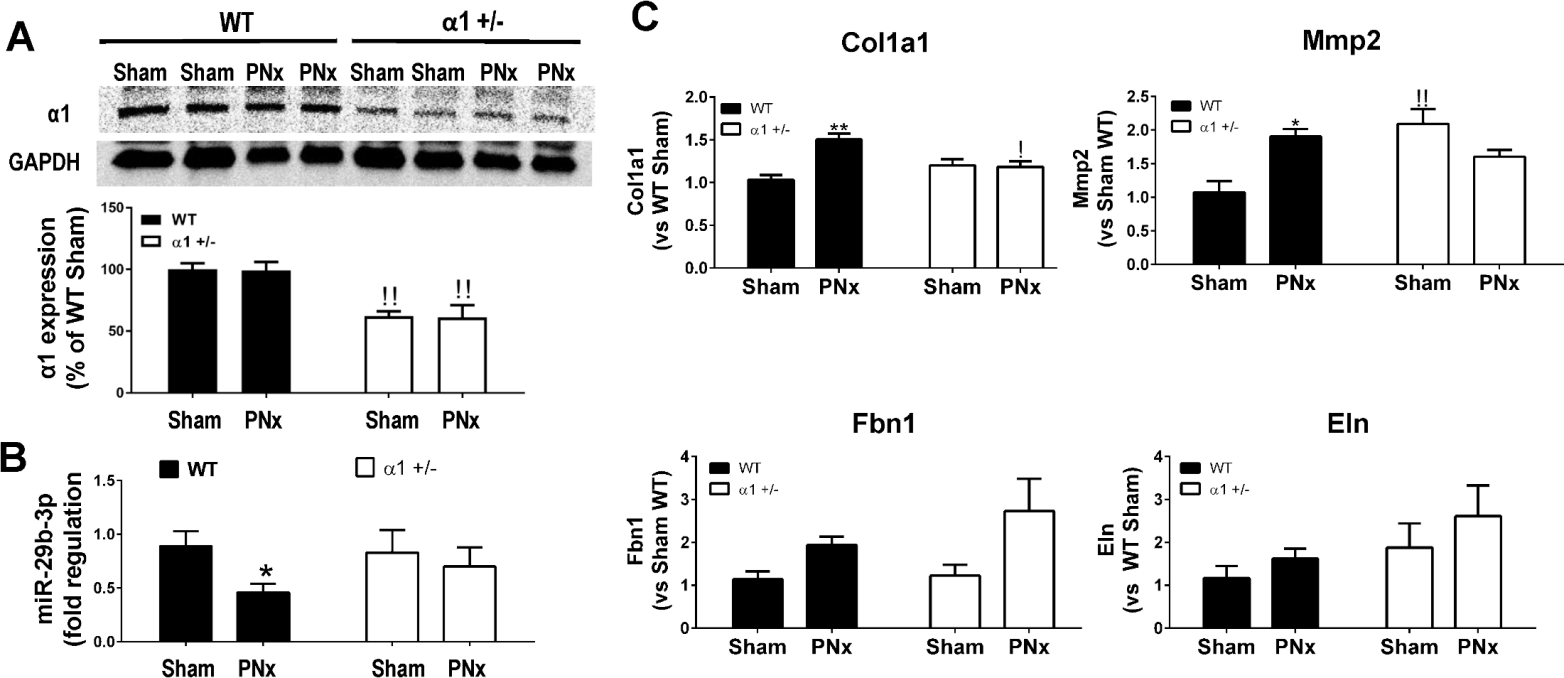
PNx-induced miR-29b-3p expression change in WT and α1+/- mice. WT and α1+/- mice were subjected to sham or 5/6^th^ partial nephrectomy (PNx) surgery and left ventricle tissue was collected at the end of 16^th^ week for RNA extraction, RT-qPCR, and Western blot analyses. **A):** Western blot of Na/K-ATPase α1 subunit expression in left ventricle tissue from different group of mice. ^!!^ indicates p<0.01 α1+/- vs WT. **B):** Expression of miR-29b-3p measured by RT-qPCR in left ventricle tissue from experimental mice. Data was analyzed using Two-Way ANOVA with GraphPad software 7.0., N = 5 in each group * indicates p<0.05 PNx vs Sham. **C):** mRNA expression of miR-29b-3p targeted genes: collagen 1A1 (Col1a1), matrix metalloproteinase-2 (Mmp2), fibrillin 1 (Fbn1), and elastin (Eln). * indicates p<0.05 PNx vs Sham; ** indicates p<0.01 PNx vs Sham. ^!!^ indicates p<0.01 α1+/- vs WT.

To test the role of the Na/K-ATPase in CKD animals, we performed PNx surgery on WT mice as well as on α1+/- mice. Cardiac tissue was collected at 16 weeks after PNx surgery. As shown in Fig 1A, cardiac Na/K-ATPase α1 subunit expression in α1+/- mice was ~40% less than that in WT mice, which is consistent with previous reports [33, 34]. When miR-29b-3p was measured by RT-qPCR using total RNA extracted from left ventricle tissue, we found that PNx decreased miR-29b-3p levels by about 2 fold in WT mice (0.89±0.14 in sham vs 0.46±0.08 in PNx, p<0.01), whereas in α1+/- mice the basal level of miR-29b-3p was slightly lower than that in WT mice, but PNx surgery caused no significant changes in miR-29b-3p expression (Fig 1B). We also measured the potential targets of miR-29b-3p such as collagen 1A1, matrix metalloproteinase-2 (Mmp-2), fibrillin 1 (Fbn1), and elastin (Eln). As shown in Fig 1C, PNx significantly increased collagen 1A1 and Mmp-2 by ~50% and 100%, respectively, in WT mice, but had no significant effect in α1+/- mice. The expression of Fbn1 and Eln was not significantly changed in either WT or α1+/- mice.

### Na/K-ATPase regulates miR-29b-3p through activation of Src and NFκB in CKD mice

**Fig 2:**
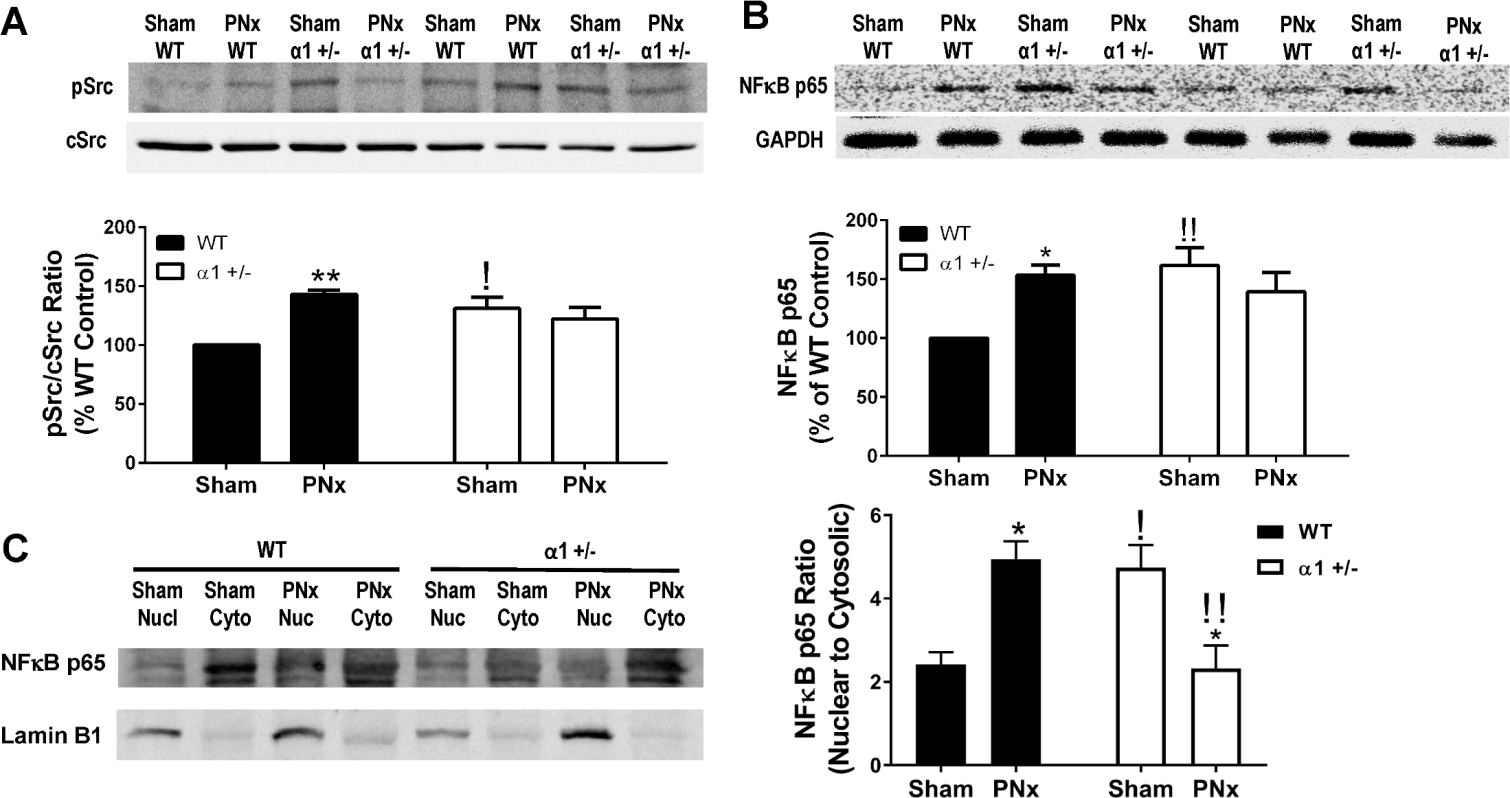
Activation of Src and NFκB in mice left ventricle tissue after PNx surgery. **A):** Western blot shows Src activation indicated by phosphorylation at Tyrosine 418 (pSrc) in left ventricle tissue, normalized to total Src levels (cSrc). Data are normalized as percentage expression of WT Sham animals. **B):** Western blots for total NFκB p65 in heart left ventricle tissue homogenates from each group. **C):** Western blots of NFκB p65 in nuclear and cytosolic fractions of isolated from left ventricle tissue. Lamin B1 is used as a nuclear marker. Nuclear/cytosolic ratio of NFκB was calculated as indicator of NFκB activation. Data were presented as mean ± SEM from 5 mice in each group. Data were analyzed using Two-Way ANOVA followed by Pairwise comparisons using Tukey’s correction for multiple comparisons, N = 4 in each group. * indicates p<0.05 PNx vs sham; ** indicates p<0.01 PNx vs Sham; ^!!^ indicates p<0.01 α1+/- sham vs WT sham or α1+/- PNx vs WT PNx.

It has been previously reported that Na/K-ATPase regulates Src [28, 34] and NFκB signaling [38, 39], while both Src and NFκB are known to regulate miR-29b-3p expression [31, 40]. We then examined these signaling pathways in both WT and α1+/- mice. As shown in Fig 2A, PNx surgery significantly increased Src phosphorylation at Tyr418 (an indication of Src activation) in the left ventricle tissue from WT mice, while it failed to induce activation of Src in α1+/- mice, albeit the basal level in α1+/- mice is higher compared to that in WT mice. PNx also caused higher expression of total NFκB p65 (Fig 2B) as well as its nuclear fraction (Fig 2C) in the left ventricle tissue from WT mice. Similarly, the basal level of NFκB p65 is higher in α1+/- mice, but PNx did not further increase the expression level or nuclear fraction of NFκB.

**Fig 3:**
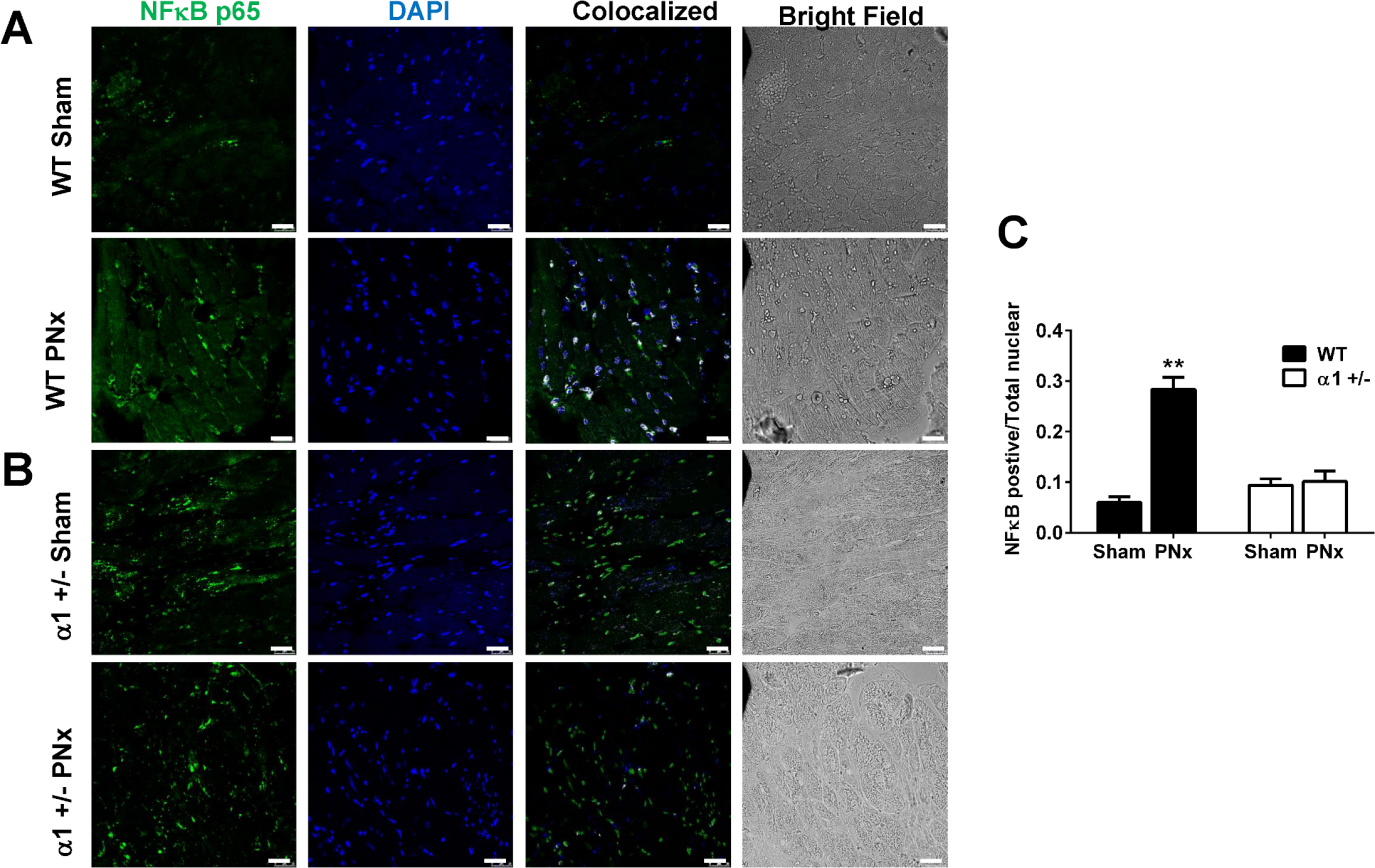
Expression and translocation of NFκB in heart tissue after PNx surgery. Left ventricle tissue slides (4 μm in thickness) from WT sham (N = 4), WT PNx (N = 5), α1+/- sham (N = 4), and α1+/- PNx (N = 4) animals were deparaffinzed and immunostained for NFκB p65. (A): Representative images from WT mice; (B): Representative images from α1+/- mice; (C): Quantification data. Colocalization of NFκB and DAPI was analyzed using ImageJ. The ratio of NFκB p65 positive nuclear and total nuclear was used for analysis by Two-Way ANOVA. ** indicates p<0.01, WT PNx vs WT sham. Scale bar is 25 μm.

We also performed immunostaining for NFκB using an anti-NFκB p65 antibody. As shown in Fig 3A, the NFκB p65 signal was weak in sham-operated WT mice, but PNx caused a significant increase in NFκB levels, especially in the smaller cells between myocytes. Most of the NFκB signal (green) was around or colocalized with the DAPI signal (purple) in these cells, indicating a nuclear transfer of NFκB p65. However, in sham-operated α1+/- mice (Fig 3B), the NFκB p65 signal in the left ventricle tissue was much higher than that in WT mice. Interestingly, in the α1+/-mice, the NFκB staining was in a punctate pattern and mostly in myocytes. PNx surgery caused no significant change in NFκB p65 expression or its location in α1+/- mice. These results of NFκB expression and activation are consistent with the observation of miR-29b-3p changes in WT and α1+/- mice, suggesting that activation of Src and NFκB may mediate the PNx-induced reduction of miR-29b-3p in WT mice.

**Fig 4:**
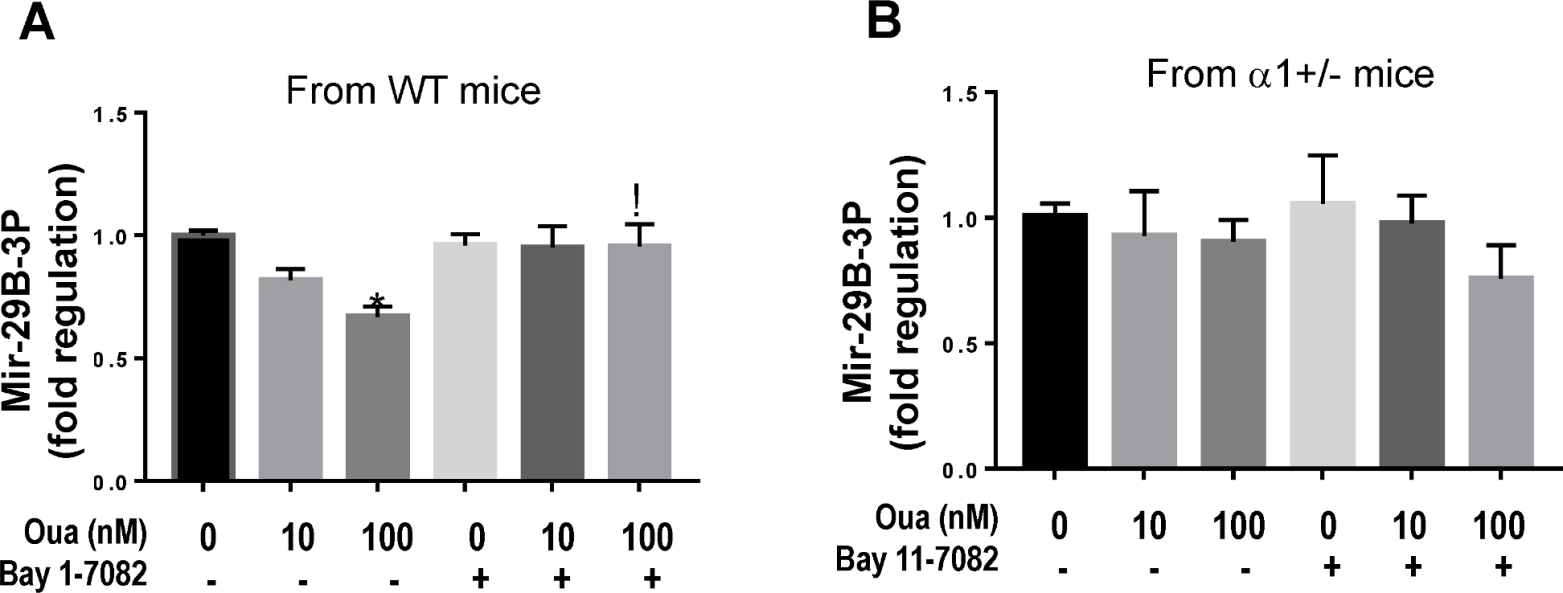
The effect of NFκB inhibitor on ouabain-induced miR-29b-3p regulation in cardiac fibroblasts isolated from WT and α1+/- mice. Isolated primary cultures of mouse cardiac fibroblasts were pretreated with 1μM of the NFκB inhibitor BAY 11–7082 for 15 min followed by ouabain (10 or 100nM) treatment for 24h. Non-treated or ouabain treatment alone without BAY 11–7082 was used as control. After treatment, cell lysates were collected. Expression of miR-29b-3p was measured using RT-qPCR in RNA isolated from cells from WT animals (**A**) and α1 +/- animals (**B**). Fibroblasts were were obtained from 4 animals in each group. Data was analyzed using One-Way ANOVA with GraphPad software 7.0. * indicates p<0.05 versus non-treated controls. ^!^ indicates p<0.05 Bay11-7082 plus ouabain vs ouabain alone.

We have previously demonstrated that Src activation is involved in Na/K-ATPase regulated miR-29b-3p in isolated cardiac fibroblasts [31]. To further determine the role of NFκB in Na/K-ATPase mediated miR-29b-3p regulation, we used isolated cardiac fibroblasts from WT as well as α1+/- mice. These fibroblasts were treated with ouabain (a specific Na/K-ATPase ligand) alone or in combination with a specific NFκB inhibitor (Bay11-7082) for 24h. Total RNA was extracted from the cell lysates and miR-29b-3p level was quantified using RT-qPCR as described previously [31]. As shown in Fig 4A, ouabain alone induced a decrease in miR-29b-3p expression in a dose-dependent manner in fibroblasts isolated from WT mice and the decrease of miR-29b-3p can be blocked by Bay11-7082, suggesting that NFκB is involved in Na/K-ATPase mediated miR-29b-3p regulation. However, in cardiac fibroblasts isolated from α1+/- mice, ouabain failed to induce the change in miR-29b-3p levels, and the NFκB inhibitor had no effect either (Fig 4B).

### Treatment with pNaKtide inhibits Src activation and antagonizes PNx-induced cardiac fibrosis by increasing miR-29b-3p expression in heart tissue from CKD mice

**Fig 5:**
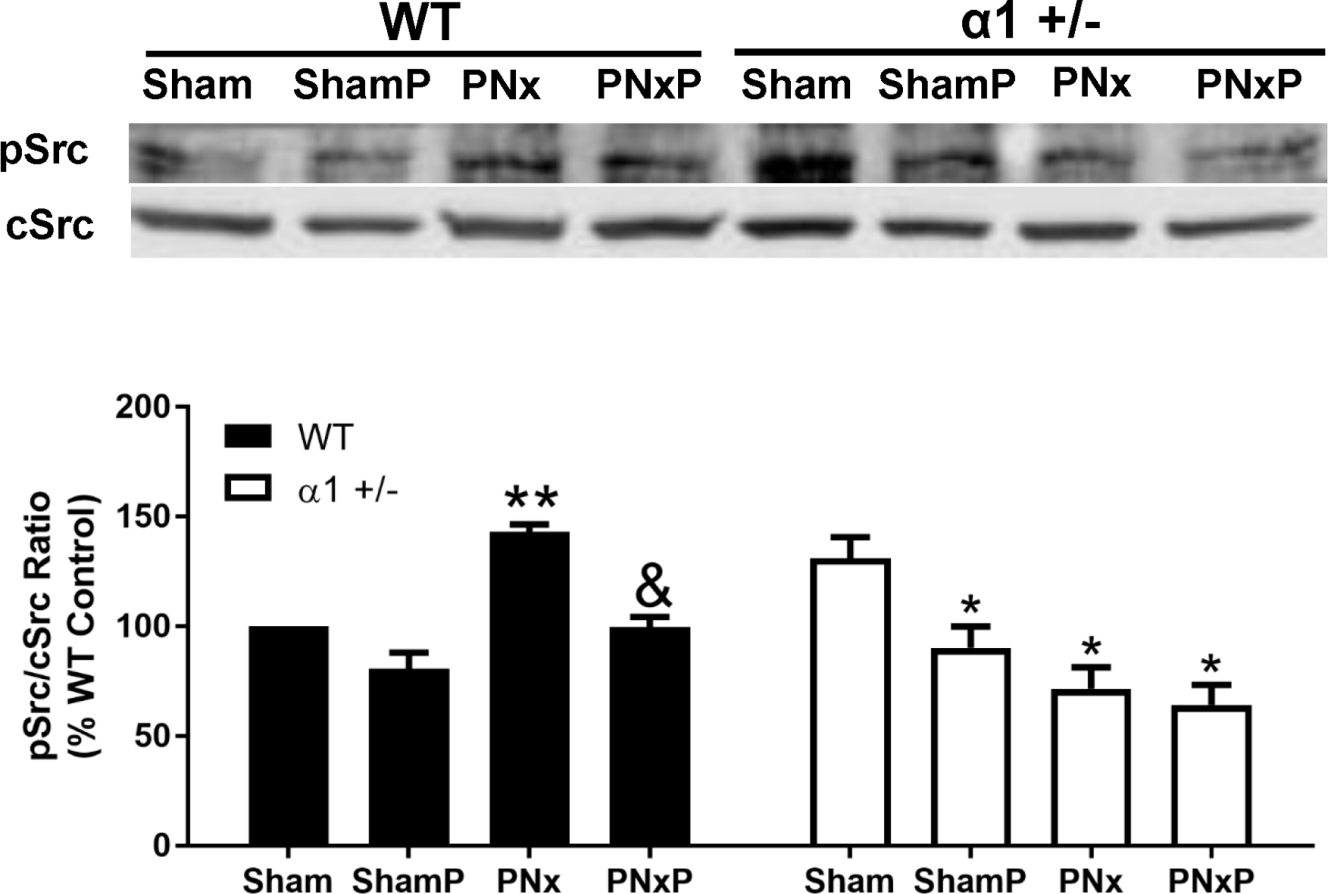
Injection of pNaKtide blocks Src activation in mice subjected to PNx surgery. Tissue homogenate obtained from left ventricle tissue were used to probe phosphor-Src (pSrc) and total Src (cSrc) using Western blot. Data were obtained from 5 animals in each group and normalized as percentage expression of WT Sham and was analyzed using Two-Way ANOVA with GraphPad software 7.0. Sham: sham-operated animals, ShamP: sham-operated animals with pNaKtide injection. PNx: PNx-operated animals, PNxP: PNx-operated animals with pNaKtide injection.* indicates p<0.05 vs Sham; ^&^ indicates p<0.05, PNxP vs PNx; ^!!^ indicates p<0.01 α1+/- PNx vs WT PNx.

The pNaKtide has been reported as an inhibitor of Na/K-ATPase related Src signaling [41, 42], which prevents ouabain-induced reduction of miR-29b-3p in isolated cardiac fibroblasts [31]. To test the effect of pNaKtide on miR-29b-3p expression and cardiac fibrosis in CKD mice, we performed PNx or sham surgery on WT mice and α1+/- mice. At 12 weeks after the PNx or sham surgery, pNaKtide was given by intraperitoneal injection at 25 mg/kg bodyweight every other week for a total of 3 injections. Mice injected with the same volume of saline were used as control. Organ collection was done at the end of the 16^th^ week after surgery. As shown in Fig 5, PNx alone caused higher Src phosphorylation at Tyr418 in left ventricle tissue from WT mice, while pNaKtide injection attenuated the PNx-induced Src phosphorylation. In α1+/- mice, the basal level of Src phosphorylation was higher compared to that in WT, but PNx was unable to further stimulate Src phosphorylation and actually decreased Src phosphorylation in these animals. Injection of pNaKtide did not change the phosphorylation state compared to PNx alone in α1+/- mice.

**Fig 6:**
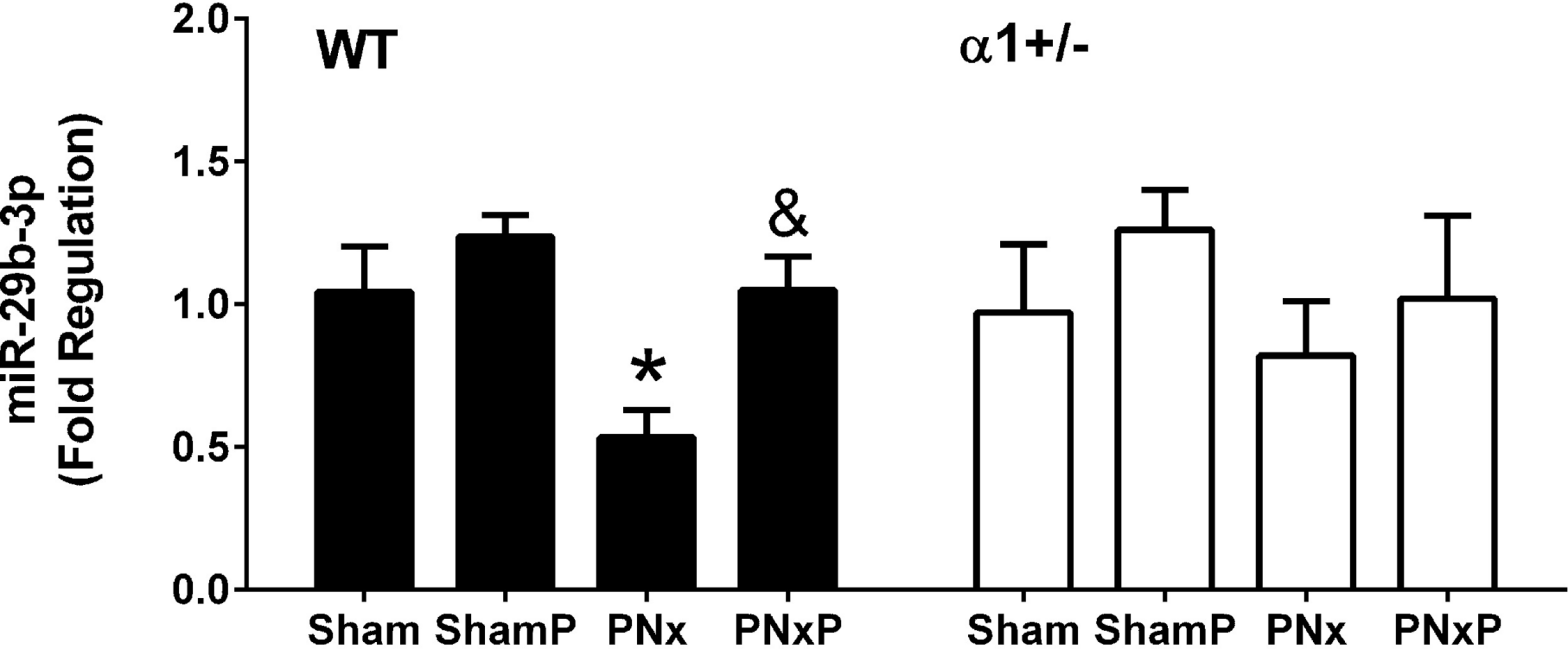
Injection of pNaKtide diminishes PNx-induced decrease in miR-29b-3p expression. Total RNA obtained from left ventricle tissue were used for RT-qPCR analyses. Expression of miR-29b-3p was presented as fold regulation relevant to WT sham animals. Data were obtained from 5 animals in each group and was analyzed using Two-Way ANOVA with GraphPad software 7.0. * indicates p<0.05 vs Sham; ^&^ indicates p<0.05 PNxP vs PNx.

Consistently, as shown in Fig 6, we found that injection of pNaKtide in WT mice significantly increased miR-29b-3p expression in heart tissue compared to that in PNx alone group. In α1+/- mice, pNaKtide slightly increased miR-29b-3p expression in heart tissue. Injection of pNaKtide in sham-operated mice caused a slight increase in miR-29b-3p expression, but it was not statistically significant.

**Fig 7:**
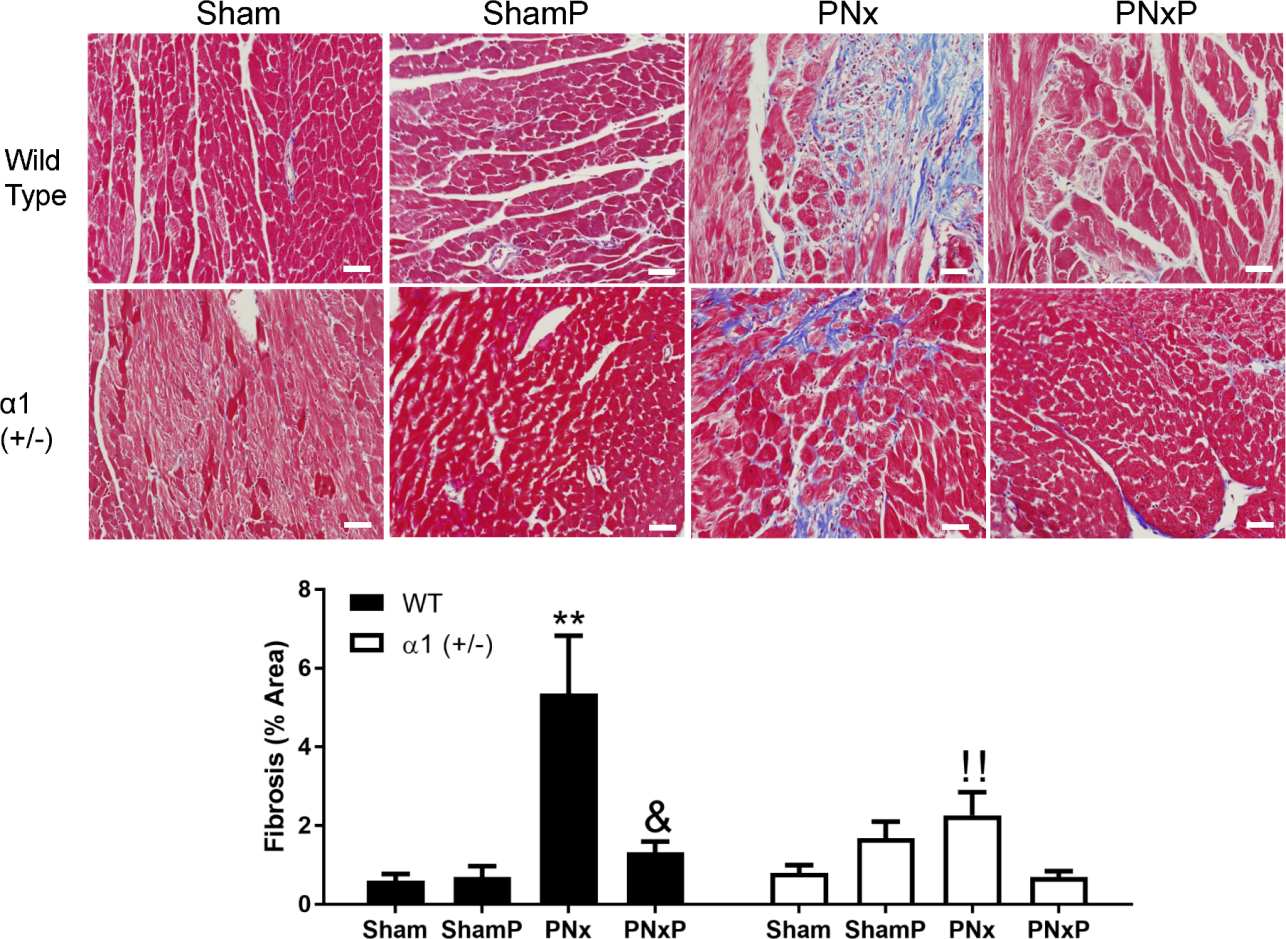
Injection of pNaKtide mitigates PNx-induced cardiac fibrosis. IP injection of 25mg/kg pNaKtide was performed biweekly started at 12^th^ week following PNx surgery and heart left ventricle tissue was collected at the end of 16^th^ week. Cardiac fibrosis was evaluated using Trichrome staining. Data are presented as Mean ± SEM, N = 10 for Sham WT, 6 for ShamP WT, 7 for PNx WT, 9 for PNxP WT, 11 for Sham α1+/-, 5 for ShamP α1+/-, 11 for PNx α1+/-, 7 for PNxP α1+/. The upper panel are representative 20x Trichrome staining. Scale bar = 25μm. The lower panel is the quantification data of fibrosis (% area). Data was analyzed using Two-Way ANOVA with GraphPad software 7.0. ** indicates p<0.01 vs Sham; ^&^ indicates p<0.05 PNxP vs PNx; ^!!^ indicates p<0.01 α1+/- PNx vs WT PNx.

To test if the above changes in Src activation and miR-29b-3p expression correlate with cardiac tissue fibrosis, formalin-fixed left ventricle tissue was analyzed using Trichrome staining as described in the Materials and Methods section. As shown in Fig 7, PNx significantly increased cardiac fibrosis by about 9-fold in WT mice (0.60% in sham vs 5.3% in PNx, p<0.01), while treatment with pNaKtide led to significantly reduced fibrosis compared to PNx alone (1.3 ± 0.3% in the PNx plus pNaKtide group vs 5.3 ± 1.5 in the PNx alone group, p<0.01) in WT mice. In α1+/- mice, the basal level of cardiac fibrosis was higher than that in WT mice, and PNx induced a milder 3-fold change in cardiac fibrosis (0.8±0.2% in sham vs 2.3±0.6% in PNx, p<0.05). Injection of pNaKtide also reduced cardiac fibrosis in α1+/- mice (0.7±0.2% in PNx plus pNaKtide group vs 2.3±0.6% in PNx alone), but did not reach statistical significance. Treatment with pNaKtide in sham-operated mice yielded no significant changes in levels of cardiac fibrosis.

### Treatment with pNaKtide ameliorates PNx-induced cardiac hypertrophy

**Fig 8:**
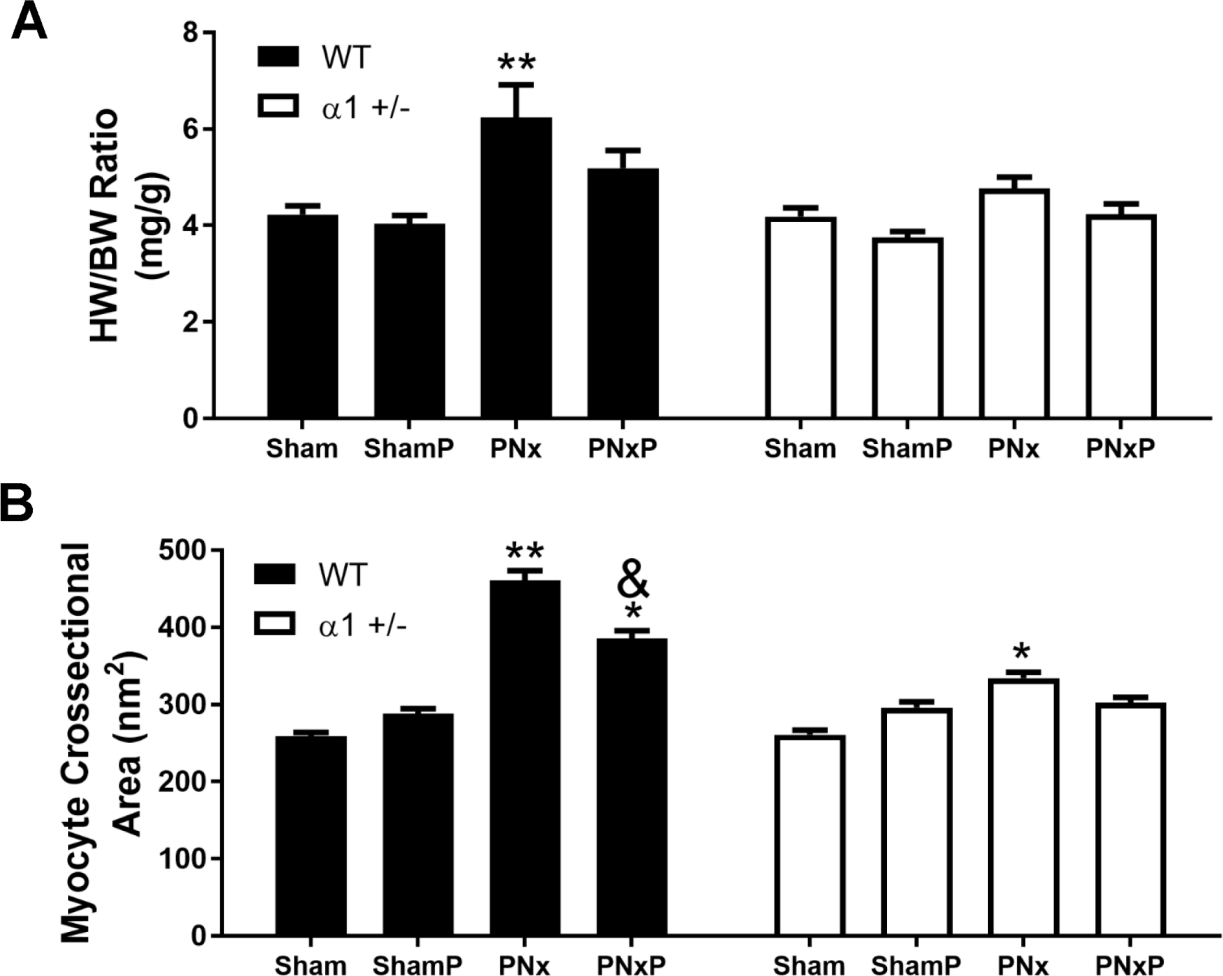
Injection of pNaKtide attenuates PNx-induced cardiac hypertrophy. **A):** Data of heart weight/body weight (HW/BW) ratio were obtained from 6–10 mice from each group. **B):** Myocytes cross sectional area were evaluated using wheat germ agglutinin (WGA) staining as described in Method section. Eight images were randomly taken from each tissue slide and 20 cells from each image were measured for their cross sectional area. Data is presented as Mean ± SEM and analyzed using Two-Way ANOVA with GraphPad software 7.0. * indicates p<0.05 vs Sham; ** indicates p<0.01 vs Sham; ^&^ indicates p<0.05 PNxP vs PNx.

In addition to its effects on fibrosis, we also examined the effect of pNaKtide on PNx-induced cardiac hypertrophy. As shown in Fig 8A, the heart weight/body weight (HW/BW) ratio of animals subjected to PNx was significantly increased compared to sham-operated animals at 16 weeks (6.2±0.7 in PNx group vs 4.2±0.2 in sham group, p<0.01). Injection of pNaKtide led to a 23.3% reduction of cardiac mass compared to PNx alone animals. In α1+/- mice, PNx also increased cardiac hypertrophy, but to a much smaller extent (4.8±0.2 in PNx vs 4.2±0.2 in sham, p>0.05). Consistent with these observations, as shown in Fig 8B, we found that PNx led to more enlarged cardiac myocytes in WT animals when the sizes of individual cardiac myocytes were analyzed using Wheat Germ Agglutinin (WGA) staining. The average cross sectional area was significantly reduced with pNaKtide injection in WT mice (461 ± 13 nm^2^ in PNx alone vs 386 ± 10 nm^2^ in pNaKtide injected animals, p<0.05). These effects of PNx or pNaKtide injection in α1+/- mice were much less pronounced.

We also evaluated other cardiac function using echocardiography. The echo data were summarized in Tables 1 and 2. We found that in WT animals there was a significant increase in diastolic dimension at 16 weeks after PNx surgery, and pNaKtide injection attenuated the increase in diastolic dimension. Ejection fraction (EF) was significantly decreased with PNx in both WT and α1+/- mice and injection of pNaKtide slightly improved EF in WT but not in α1 +/- mice.

**Table 1:** Echocardiographic data in WT mice 16 weeks following PNx or Sham surgery.

Variable	Sham (n = 10)	ShamP (n = 6)	PNx (n = 7)	PNxP (n = 9)
Heart Rate (BPM)	461 ± 14	511 ± 22	438 ± 12	458 ± 19
EDV (μL)	37.2 ± 2.2	33.6 ± 4.1	52.0 ± 4.5*	47.3 ± 4.3
ESV (μL)	18.1 ± 2.4	17.4 ± 2.8	33.0 ± 5.0*	24.7 ± 4.5
EF (%)	61.5 ± 5.0	48.3 ± 4.4	40.8 ± 4.6*	50.0 ± 3.0
IVSWT at Diastole (mm)	0.93 ± 0.07	0.86 ± 0.08	0.91 ± 0.09	0.92 ± 0.10
PWT at Diastole (mm)	0.94 ± 0.06	0.85 ± 0.08	0.78 ± 0.12	1.00 ± 0.10
LVMI	4.1 ± 0.3	3.4 ± 0.2	5.3 ± 0.9	4.6 ± 1.0

IVSWT: interventricular septal wall thickness; PWT: posterior wall thickness; WTI: wall thickness index ([PWT + IVSWT]/DD). Left ventricle mass index (LVMI). Data presented as Mean ±SEM.

*, indicates p<0.05 different from sham. Sham: sham-operated control group; ShamP: sham-operated group that received pNaKtide injection; PNx: mice subjected to PNx surgery; PNxP: mice subjected to PNx surgery and received pNaKtide injection.

**Table 2:** Echocardiographic data in α1+/- mice 16 weeks following PNx or Sham surgery.

Variable	Sham (n = 11)	ShamP (n = 5)	PNx (n = 11)	PNxP (n = 7)
Heart Rate (BPM)	436±52	504±26*	458±34	472±28
EDV (μL)	31.6±2.7	40.1±5.6	43.8±2.4*	39.0±4.4
ESV (μL)	14.1±1.8	18.9±3.5	23.3±1.8*	23.2±3.3
EF (%)	56.5±2.8	53.7±3.5	47.1±2.6	41.9±3.6*
IVSWT at Diastole (mm)	0.86±0.05	0.75 ± 0.05	0.84 ± 0.05	0.93 ± 0.07
PWT at Diastole (cm)	0.85±0.04	0.76 ± 0.10	0.81 ± 0.04	0.88 ± 0.05
LVMI	3.2 ±0.2	3.2 ± 0.6	4.0 ± 0.3	3.9 ± 0.3

IVSWT: interventricular septal wall thickness; PWT: posterior wall thickness; WTI: wall thickness index ([PWT + IVSWT]/DD). Left ventricle mass index (LVMI). Data presented as Mean ±SEM.

*, indicates p<0.05 different from sham. Sham: sham-operated control group; ShamP: sham-operated group that received pNaKtide injection; PNx: mice subjected to PNx surgery; PNxP: mice subjected to PNx surgery and received pNaKtide injection.

## Discussion

Multiple studies have found a significant role of anti-fibrotic microRNAs in the prevention of cardiac fibrosis [3–6, 8, 10]. Our previous data showed that prolonged activation of Na/K-ATPase signaling contributes to cardiac fibrosis in uremic cardiomyopathy [43]. More recently, we found that mimicry of miR-29b-3p can prevent CTS-induced collagen synthesis in cardiac fibroblasts [31], indicating that an increase in miR-29b-3p expression is a potential therapeutic treatment strategy for fibrosis-related diseases. In addition, our *in vitro* data showed that pNaKtide treatment prevented excessive collagen synthesis by restoring endogenous miR-29b-3p expression to levels close to non-treated controls [31]. The current study further showed the same effect of pNaKtide *in vivo* as well as its potential in attenuating PNx-induced cardiac fibrosis and hypertrophy.

Mechanistically, the results from this study demonstrate that CKD induces activation of Na/K-ATPase-mediated Src and its downstream target NFκB. As a transcription factor, NFκB can form a complex with Sp1 and HDAC and directly bind to the regulatory sequence of the miR-29b-3p gene, causing decreased expression of miR-29b-3p [40]. Disrupting the Na/K-ATPase-related signaling and inhibition of Src activation by pNaKtide increased miR-29b-3p expression in heart tissue and thus attenuated cardiac fibrosis in these animals. The data from α1+/- mice also demonstrated that miR-29b-3p regulation requires Na/K-ATPase signaling activation. Reduction of Na/K-ATPase α1 caused a deficiency in Src and NFκB activation, and CKD caused no significant changes on miR-29b-3p expression in these animals. However, an interesting observation is that even though the basal level of Src phosphorylation and NFκB activation is higher in heart tissue from α1+/- mice compared to their WT littermates, the miR-29b-3p expression was not significantly different between these animals. The specific mechanism for this phenomenon is still elusive. Our previous studies also showed that activation of Src by ouabain treatment in pig kidney proximal tubule cells induced cell proliferation, whereas in Na/K-ATPase-reduced cell lines ouabain caused cell growth inhibition or death despite the increased basal level of Src phosphorylation in these cells [44]. Similarly, we demonstrated that the increased basal level of Src phosphorylation cannot prevent cardiac cell apoptosis in α1+/- mice when infused with marinobufagenin, another specific Na/K-ATPase ligand [34]. A possible explanation is that Na/K-ATPase reduction-induced Src activation is an adaptive process. This adaptive change in Src phosphorylation is required to maintain the normal cellular function, but it cannot further respond to extrinsic stress such as CKD or ouabain treatment. It could also be due to the compartmentalization of these signaling molecules.

Reduction of Na/K-ATPase in α1+/- mice may also cause the change in Na^+^ and K^+^ transporting activity, especially in the kidney, which only expresses the α1 subunit of Na/K-ATPase. However, the measurement of plasma Na^+^ concentration (148.8±0.9 mM, n = 5) from these animals was not significantly different from WT animals (148.4±1.9 mM, n = 5), suggesting that the ion transporting activity may be compensated by increased per unit activity of Na/K-ATPase. This is in line with our previous observation that reduction of Na/K-ATPase α1 subunit by 40% resulted in only about 20% decrease in ion transporting activity in pig kidney epithelial cell line [45].

In addition, we found that even though Na/K-ATPase reduction in α1+/- mice caused a deficiency in Na/K-ATPase signaling and prevented miR-29b-3p dysregulation, it did not completely block PNx-induced cardiac fibrosis, suggesting that other pathways also contribute to the formation of cardiac fibrosis in this model. Our previous studies showed that cardiac apoptosis increased in α1+/- mice subjected to PNx surgery or MBG infusion [34, 35]. These apoptotic events may cause the release of cytokines that stimulate fibrotic changes in the tissue. These results also suggest that the reduction of Na/K-ATPase may not be an appropriate strategy to reduce CKD-related cardiac fibrosis. In fact, a decrease in Na/K-ATPase has been reported in heart failure patients and the amount of Na/K-ATPase was correlated with the ejection fraction in these patients [46, 47].

In summary, the current study demonstrate that Na/K-ATPase signaling is an important mediator that regulates miR-29b-3p, which contributes to the formation of cardiac fibrosis in the setting of CKD.

## Supporting information

S1 Fig The whole membrane Western blot image for Fig 1A. Na/K-ATPase α1 expression in WT and α1+/- mice.
(PDF)

S2 Fig The whole membrane Western blot image for Fig 2A. PNx-induced Src phosphorylation at Tyr 418 (pSrc) in left ventricle tissue from WT and α1+/- mice.
(PDF)

S3 Fig The whole membrane Western blot image for Fig 2B. PNx-induced change in NFκB p65 expression in left ventricle tissue from WT and α1+/- mice.
(PDF)

S4 Fig The whole membrane Western blot image for Fig 2C. PNx-induced NFκB p65 nuclear translocation in left ventricle tissue from WT and α1+/- mice.
(PDF)

S5 Fig The whole membrane Western blot image for Fig 5. The effect of pNaKtide on PNx-induced Src phosphorylation at Tyr 418 (pSrc) in left ventricle tissue from WT and α1+/- mice.
(PDF)
