# Supplementary figures and images for: Na/K-ATPase signaling mediates miR-29b-3p regulation and cardiac fibrosis formation in mice with chronic kidney disease
Source: PLoS One. 2018 May 18;13(5):e0197688. doi: 10.1371/journal.pone.0197688 (PMC5959191; doi:10.1371/journal.pone.0197688)

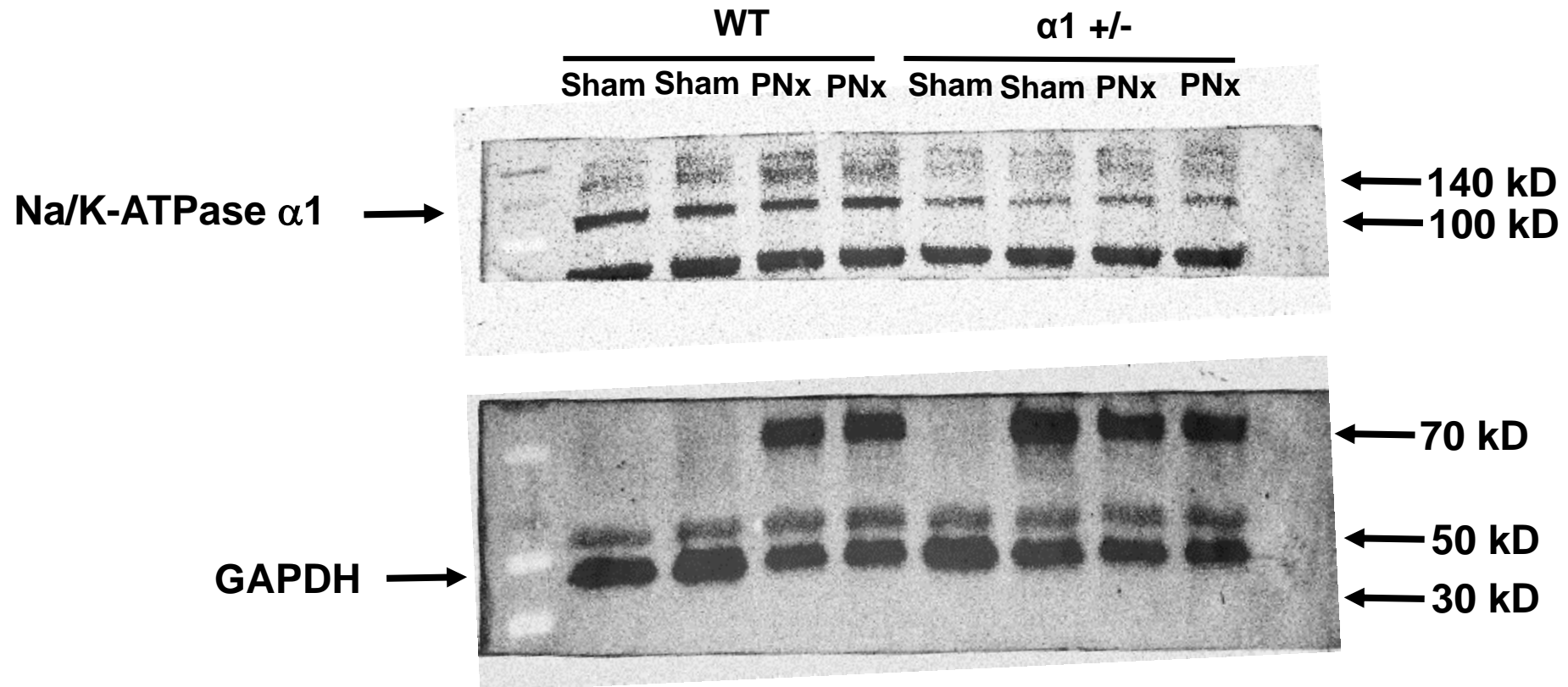

S1 Fig.

Supplement: S1 Fig — Na/K-ATPase α1 expression in WT and α1+/- mice. (PDF) [file pone.0197688.s001.pdf]

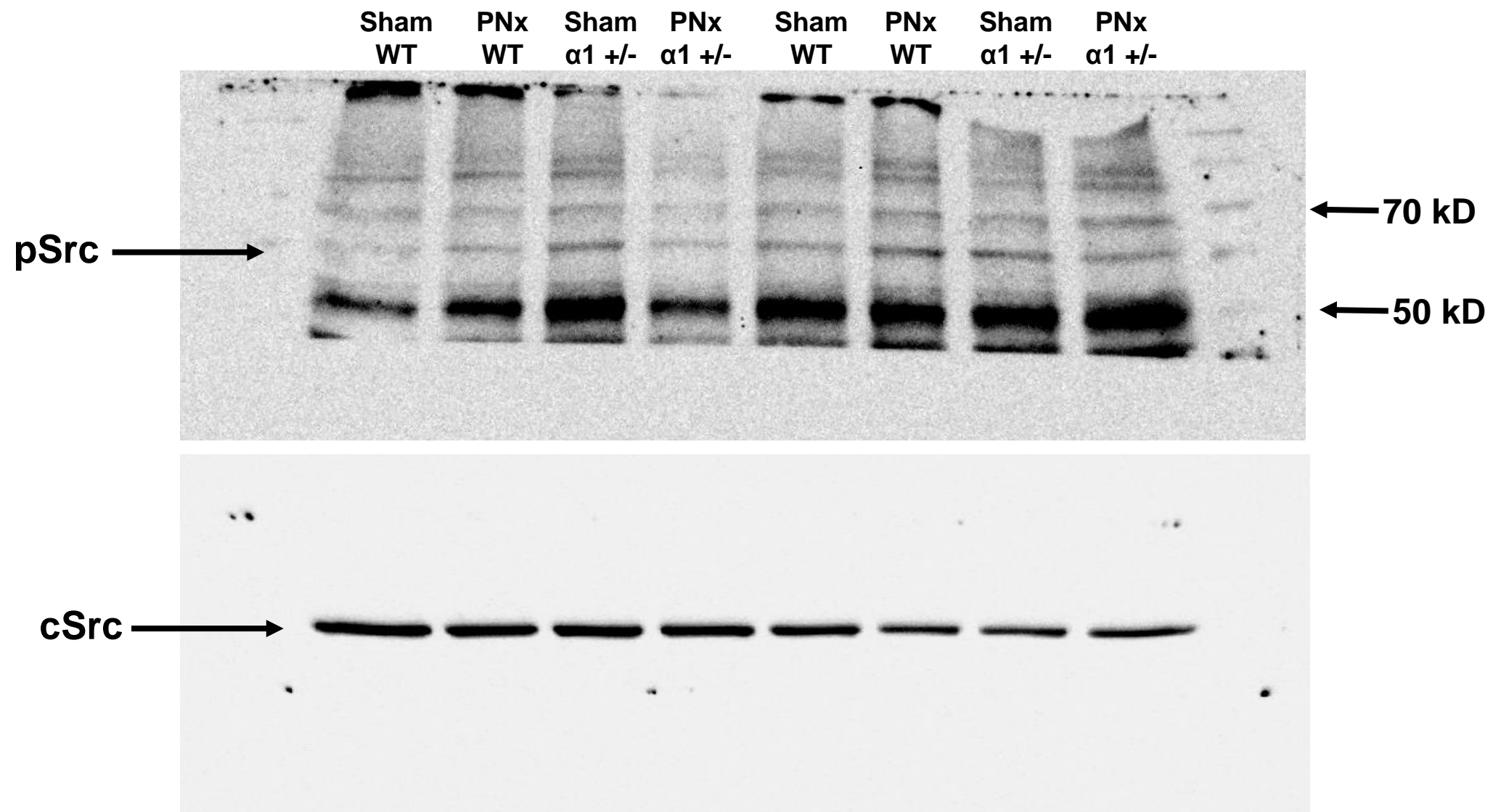

S2 Fig.

Supplement: S2 Fig — PNx-induced Src phosphorylation at Tyr 418 (pSrc) in left ventricle tissue from WT and α1+/- mice. (PDF) [file pone.0197688.s002.pdf]

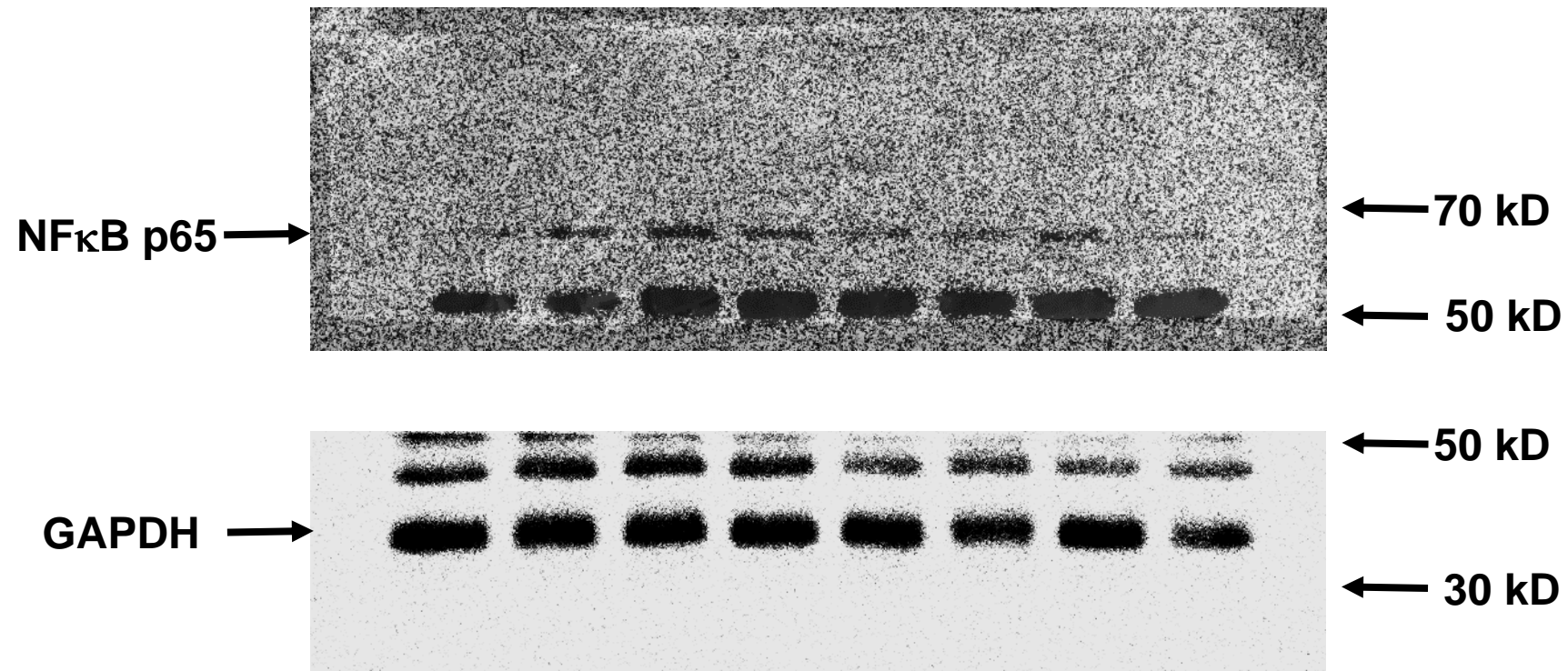

**S3 Fig.**

Supplement: S3 Fig — PNx-induced change in NFκB p65 expression in left ventricle tissue from WT and α1+/- mice. (PDF) [file pone.0197688.s003.pdf]

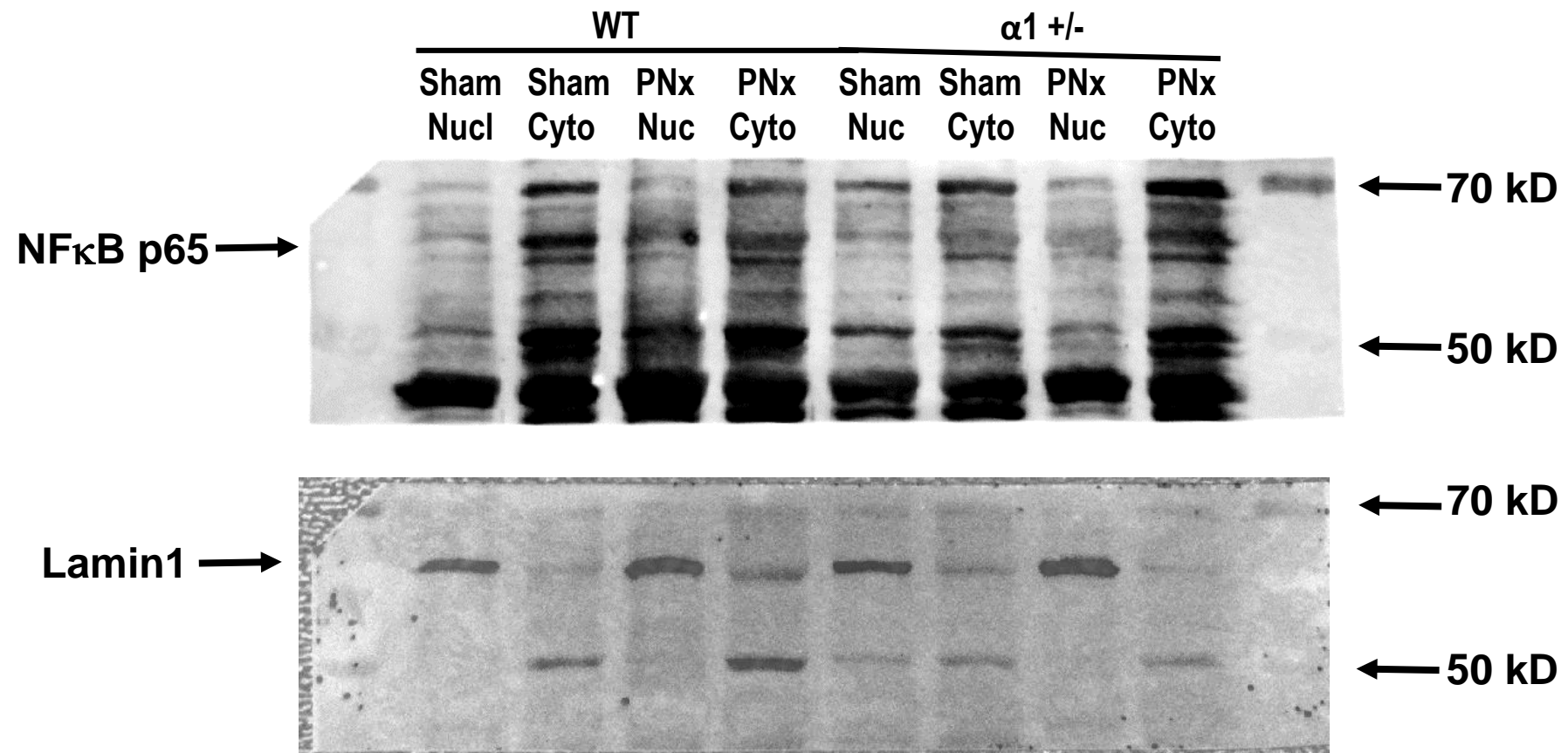

S4 Fig.

Supplement: S4 Fig — PNx-induced NFκB p65 nuclear translocation in left ventricle tissue from WT and α1+/- mice. (PDF) [file pone.0197688.s004.pdf]

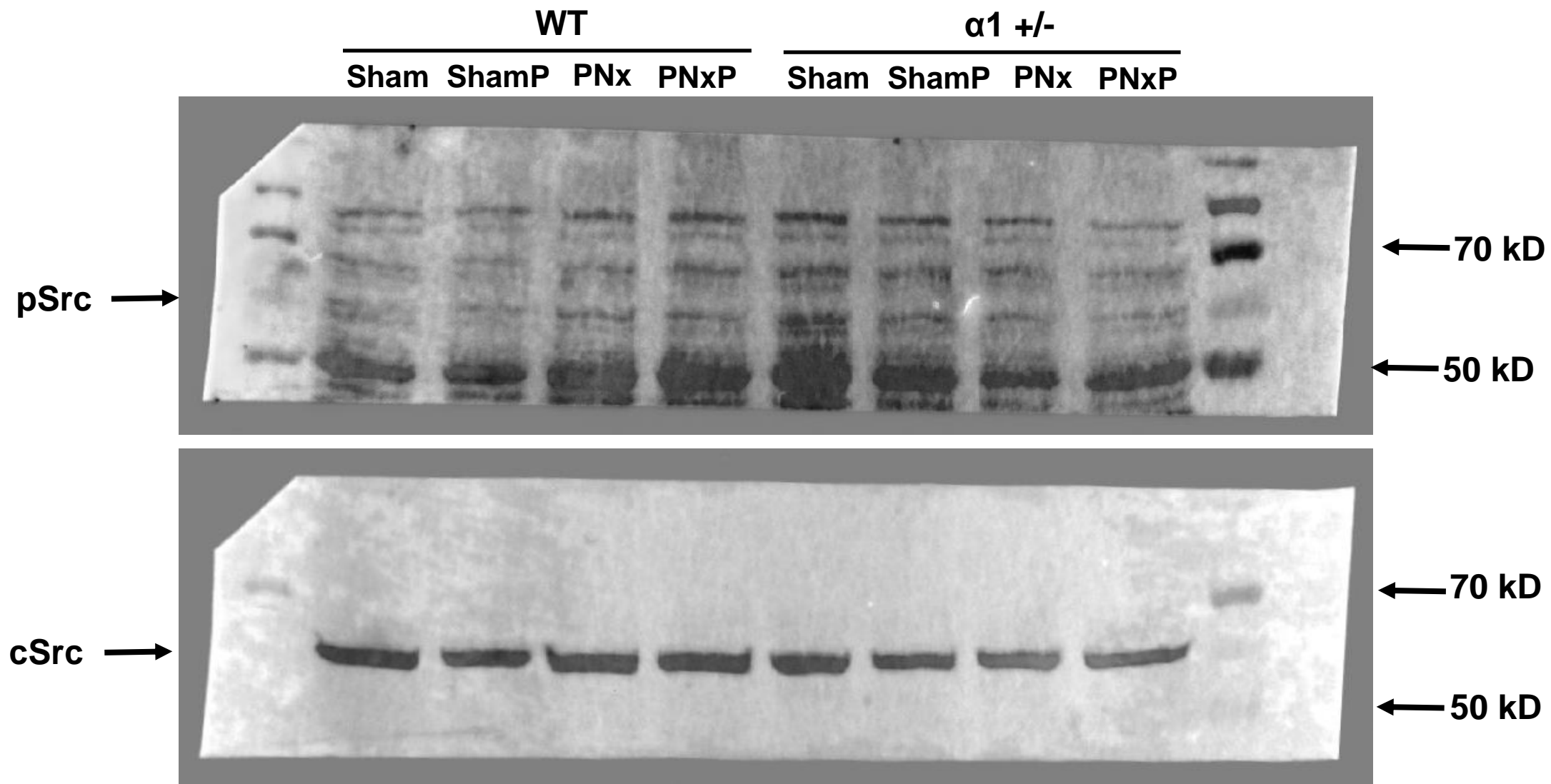

S5 Fig.

Supplement: S5 Fig — The effect of pNaKtide on PNx-induced Src phosphorylation at Tyr 418 (pSrc) in left ventricle tissue from WT and α1+/- mice. (PDF) [file pone.0197688.s005.pdf]
